# Preoperative low tri-iodothyronine concentration is associated with worse health status and shorter five year survival of primary brain tumor patients

**DOI:** 10.18632/oncotarget.14376

**Published:** 2016-12-30

**Authors:** Adomas Bunevicius, Vytenis Pranas Deltuva, Sarunas Tamasauskas, Timothy Smith, Edward R. Laws, Robertas Bunevicius, Giorgio Iervasi, Arimantas Tamasauskas

**Affiliations:** ^1^ Department of Neurosurgery, Lithuanian University of Health Sciences, Kaunas, Lithuania; ^2^ Neuroscience Institute, Lithuanian University of Health Sciences, Kaunas, Lithuania; ^3^ Department of Neurosurgery, Brigham and Women's Hospital, Boston, Massachusetts, USA; ^4^ Behavioural medicine institute, Lithuanian University of Health Sciences, Palanga, Lithuania; ^5^ CNR Institute of Clinical Physiology, Pisa, Italy

**Keywords:** tri-iodothyronine, thyroid, glioma, meningioma, survival

## Abstract

**Background:**

Low tri-iodothyronine syndrome is associated with worse prognosis of severely ill patients. We investigated the association of thyroid hormone levels with discharge outcomes and 5-year mortality in primary brain tumor patients.

**Methods:**

From January, 2010 until September, 2011, 230 patients (70% women) before brain tumor surgery were evaluated for cognitive (Mini mental State Examination; MMSE) and functional (Barthel index; BI) status, and thyroid function profile. The Low triiodothyronine syndrome was defined as triiodothyronine concentration below the reference range. Unfavorable discharge outcomes were Glasgow outcome scale score of ≤3. Follow-up continued until November, 2015.

**Results:**

Seventy-four percent of patients had Low triiodothyronine syndrome. Lower total tri-iodothyronine concentrations were associated with lower MMSE (p=.013) and BI (p=.023) scores independent of age, gender and histological diagnosis. Preoperative Low tri-iodothyronine syndrome increased risk for unfavorable discharge outcomes adjusting for age, gender and histological diagnosis (OR=2.944, 95%CI [1.314-6.597], p=.009). In all patients, lower tri-iodothyronine concentrations were associated with greater mortality risk (p≤.038) adjusting for age, gender, extent of resection, adjuvant treatment and histological diagnosis. The Low tri-iodothyronine syndrome was associated with greater 5-year mortality for glioma patients (HR=2.197; 95%CI [1.160-4.163], p=.016) and with shorter survival (249 [260] vs. 352 [399] days; p=.029) of high grade glioma patients independent of age, gender, extent of resection and adjuvant treatment.

**Conclusions:**

The Low tri-iodothyronine syndrome is common in brain tumor patients and is associated with poor functional and cognitive status, and with worse discharge outcomes. The Low tri-iodothyronine syndrome is associated with shorter survival of glioma patients.

## INTRODUCTION

The mortality associated with brain tumors remains high and largely depends on histological diagnosis, patient clinical status and extent of surgical removal [1, 2]. Novel biomarkers are expected to aid in early diagnosis, assist in monitoring tumor recurrence and response to therapy, improve prognostication, and contribute towards developing novel intervention strategies of brain tumors [3, 4]. The Low triiodothyronine syndrome is the most common pattern of abnormal thyroid hormone metabolism in critical illness that is characterized by decreased peripheral concretions of triiodothyronine, which is the most potent thyroid gland hormone [5, 6]. The Low triiodothyronine syndrome is highly prevalent among patients suffering from neurological disorders and has been linked to more severe health status and to worse prognosis independently of disease severity [7–9]. Lower triiodothyronine concentrations were also linked to more aggressive behavior of cancer cells [10, 11] and with shorter survival of lung cancer patients [12]. However, the clinical significance of the Low triiodothyronine syndrome in brain tumor patients remains understudied [13–16]. In 90 patients with various histological diagnoses of brain tumors we have documented that 54% of patients had the Low triiodothyronine syndrome that was associated with an 8-fold increase in risk for unfavorable discharge outcomes and with greater depressive and anxiety symptom severity [15]. However there are no studies investigating long-term prognostic value of the Low triiodothyronine syndrome in primary brain tumor patients.

The goal of the present study was to evaluate the association of the Low triiodothyronine syndrome and other HPT-axis hormone concentrations with disease severity, postoperative outcomes and 5-year prognosis of brain tumor patients.

## RESULTS

In 93 (40%) patients the diagnosis was meningioma; in 49 (21%), high-grade glioma; in 36 (16%) pituitary adenoma; in 22 (10%), low-grade glioma; and in 14 (6%) acoustic neuroma (Table 1). Seventeen percent of patients were operated for recurrent tumor, 87% underwent gross total tumor removal and 27% of patients received adjuvant therapy following surgery.

**Table 1:** Baseline characteristics of the study patients

Characteristic	Median (IQR) and mean±SD for continuous variables and number (%) for categorical variables
**Age**	58 (21); 55.9±14.4
**Gender**	
Men	70 (30%)
Women	160 (70%)
**Histological diagnosis of brain tumor**	
High grade glioma	49 (21%)
Low grade glioma	22 (10%)
Meningioma	93 (40%)
Pituitary adenoma	36 (16%)
Acoustic neuroma	14 (6%)
Other histological diagnosis ^A^	16 (7%)
**Recurrent brain tumor**	38 (17%)
**Admission Mini Mental State Examination** (n=94)	
Score	27 (4); 26.23±3.82
Unfavorable cognitive status (score <24)	19 (8%)
**Admission Barthel index** (n=105)	
Score	100 (0); 95.33±13.56
Unfavorable functional status (score <90)	14 (6%)
**Thyroid hormone serum concentrations**	
TSH (μIU/ml)	.70 (.98); 1.04±1.28
Free triiodothyronine (pg/ml)	1.59 (.72); 1.66±.50
Total triiodothyronine (ng/dl)	69.58 (26.21); 73.59±43.43
Total thyroxine (μg/dl)	6.90 (2.20); 7.06±1.76
Free thyroxine (pg/ml)	10.49 (4.58); 10.89±3.47
Reversed triiodothyronine (ng/ml)	.42 (.26); .44±.19
**Low free triiodothyronine concentrations** (<2 pg/ml)	170 (74%)
**Low total triiodothyronine concentrations** (<70 ng/dl)	115 (50%)
**Extent of brain tumor removal**	
Gross total	201 (87)
Subtotal	26 (11)
Biopsy	3 (2)
**Discharge Glasgow outcome scale**	
Score	4 (1); 4.07±.96
Poor discharge outcome (score ≤3)	43 (19)
**Adjuvant therapy**	62 (27%)
**Chemotherapy**	22 (9%)
**Radiotherapy**	61 (26%)
**Follow up** (years)	5.08 (.73); 5.07±0.44
**Overall survival rate**	150 (80%)
**Survival time** (in years)	3.71±1.92
**Five year mortality rate**	
All-cause	80 (35%)
Brain tumor related	60 (36%)

TSH, Thyroid stimulating hormone; T4, thyroxine and T3, triiodothyronine

^A^ CNS lymphoma (3), craniopharyngioma (2), medulloblastoma (2), choroid plexus papilloma (1), angioreticuloma (1), hemangiopericytoma (1), pineal gland tumor (1), solitary fibrous tumor (1), hemangioma (1) and hemangioblastoma (1)

### Association with BT histological diagnosis and health status

Free and total triiodothyronine concentrations below the reference range were recorded in 74% and 50% of patients, respectively. High-grade glioma patients, relative to patients with other histological diagnoses, had significantly lower triiodothyronine (free and total) concentrations, greater rT3 concentrations, and greater prevalence of low free triiodothyronine concentrations (p≤.002) (Table 2).

**Table 2:** HPT-axis hormone concentrations and prevalence of low T3 syndrome as a function of histological diagnosis of brain tumor ^A^

	Brain tumor histological diagnosis						F (p) or	X^2^ (p)
	High grade glioma	Low grade glioma	Meningioma	Pituitary adenoma	Acoustic neuroma	Other		
TSH (μIU/ml)	.62 (.74)	1.24 (.82)	.63 (1.08)	.93 (1.23)	.45 (1.07)	.69 (.93)	6.86 (.231)	-
Free triiodothyronine (pg/ml)	1.48 (.53)	1.51 (.61)	1.54 (.73)	1.97 (.74)	1.90 (.92)	1.38 (.74)	**26.14 (<.001)**	-
Total triiodothyronine (ng/dl)	57.93 (23.01)	73.19 (24.31)	66.44 (22.80)	83.41 (26.11)	78.42 (29.41)	62.97 (30.02)	**28.29 (<.001)**	-
Free thyroxine (pg/ml)	11.00 (4.04)	9.92 (3.89)	10.52 (4.83)	9.14 (4.45)	11.53 (4.27)	10.84 (4.60)	**12.95 (.024)**	-
Total thyroxine (μg/dl)	6.60 (2.10)	6.60 (1.95)	7.05 (2.57)	6.30 (2.10)	7.75 (3.62)	7.00 (1.80)	8.11 (.15)	-
Reversed triiodothyronine (ng/ml)	.49 (.25)	.42 (.17)	.46 (.25)	.26 (.15)	.37 (.26)	.45 (.46)	**42.78 (<.001)**	-
Low free triiodothyronine concentrations (<2 pg/ml)	43 (88%)	18 (86%)	69 (74%)	19 (53%)	7 (50%)	14 (88%)	-	**20.57 (.001)**
Low total triiodothyronine concentrations (<70 ng/dl)	30 (61%)	9 (43%)	54 (58%)	8 (22%)	5 (36%)	9 (60%)	-	**18.16 (.003)**

^A^ Kruskal Wallis test for continuous variables and Pearson Chi-squared test for categorical variables.

Data presented as median (interquartile range)

P-values of <.05 are in bold.

There was a positive correlation between total triiodothyronine concentrations and MMSE score (rho=.234, p=.023), but not BI score (p=.064). In univariate regression analyses, lower free and total triiodothyronine concentrations, and low total triiodothyronine concentration were associated with greater odds for unfavorable functional and cognitive status (Table 3). After adjusting for age, gender and histological diagnosis, lower free and total triiodothyronine concentrations remained associated with greater odds for unfavorable cognitive status (p=.039 and p=.013, respectively), and lower total triiodothyronine concentrations, with greater odds for unfavorable functional status (p=.023).

**Table 3:** HPT-axis hormone concentrations and ratios as a function of admission poor functional status (n=14/105; 6%) and poor cognitive status (n=19/94; 8%)

	Unfavorable functional status ^A^		Unfavorable cognitive status ^B^	
	Univariate	Multivariate ^C^	Univariate	Multivariate ^C^
	Odds ratio, 95% confidence interval, p-value			
Free triiodothyronine (pg/ml)	.273 (.079-.943), .04	ns	.235 (.073-.755), .015	.276 (.081-.938), .039
Total triiodothyronine (ng/dl)	.946 (.910-.983), .005	.953 (.914-.993), .023	.955 (.924-.987), .006	.958 (.926-.991), .013
Low total triiodothyronine concentrations (<70 ng/dl)	3.487 (1.017-11.954), .047	ns	3.075 (1.054-8.974), .040	ns

Ns, not significant

^A^ – Barthel index score < 90

^B^ – Mini Mental State Examination score <24

^C^ – Adjusted for age, gender and histological diagnosis (enter method).

### Association with discharge outcome

A greater discharge GOS score was associated with greater free and total triiodothyronine (rho=.271, p<.001 and rho=.292, p<.001, respectively) concentrations and with lower rT3 concentrations (rho=-.223, p=.001).

In univariate regression analyses, unfavorable discharge outcome was associated with lower total and free triiodothyronine concentrations, with greater rT3 concentrations, and with the Low triiodothyronine syndrome (p-values ≤.03). After adjusting for age, gender and histological diagnosis, lower free (OR=.232, 95%CI [.088-.612], p=.003) and total (OR=.967, 95%CI [.944-.990], p=.005) triiodothyronine concentrations and Low triiodothyronine syndrome (OR=2.944, 95%CI [1.314-6.597], p=.009) remained associated with unfavorable discharge outcome.

### Association with mortality

There were 80 (35%) deaths during the 5.07±0.44 year follow-up, of which 60 (36%) deaths were brain tumor related. There were 48 (98%) deaths in high grade glioma patients; 3 (14%), in low grade glioma patients; 20 (22%), in meningioma patients; 3 (8%), in pituitary adenoma patients; 2 (14%), in acoustic neuroma patients; and 4 (25%), in patients with other histological diagnoses.

**Figure 1:**
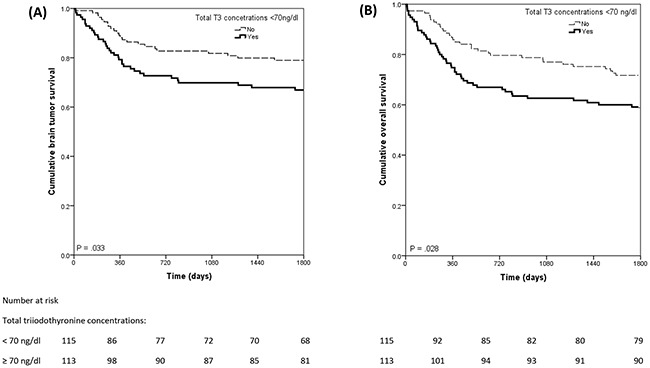
Kaplan Meier curves of overall survival (A) and brain tumor survival (B) in total sample as a functional low total tri-iodothyronine concentrations

In all patients, Kaplan-Meier estimates demonstrated worse 5-year overall and brain tumor survival rates among patients with low total triiodothyronine concentrations relative to patients with normal total triiodothyronine concentrations (59% vs. 72%, respectively; *X^2^*= 4.821, p=.028; and 69% vs. 80%, respectively, *X^2^*=4.546, p=.033) (Figure 1). Patients with low free triiodothyronine concentrations relative to patients with normal free triiodothyronine concentrations also had worse 5-year overall (62% vs. 76%; *X^2^* =4.026, p= .045) and brain tumor survival rates (69% vs. 88%; *X^2^* =7.400, p=.007).

**Figure 2:**
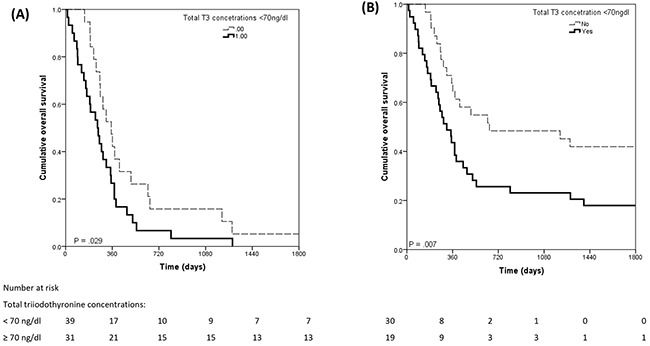
Kaplan Meier curves of overall survival in glioma patients (A) and in high-grade glioma patients only (B)

In glioma patients, all deaths were attributed to brain tumor. Low total triiodothyronine concentrations, relative to normal total triiodothyronine concentrations, were associated with worse 5-year overall survival rates for high grade and low grade glioma patients (35% vs. 65%; *X^2^*=6.370, p=.012) and with shorter overall survival time for high grade glioma patients (249 [260] vs. 352 [399] days, *X^2^* = 4.772, p=.029) (Figure 2). Low free triiodothyronine concentrations were not associated with 5-year survival of glioma patients (p = .93).

In univariate Cox regression models for the total patient sample, low total and free triiodothyronine concentrations were associated with elevated 5-year overall mortality risk (HR=1.646, 95%CI [1.050-2.580], p=.03 and HR=1.791, 95% [1.005-3.192], p=.048, respectively) and brain tumor related mortality risk (HR=1.754; 95%CI [1.039-2.960], p=.035 and HR=2.848-6.272, 95%CI [1.294-6.272], p=.009, respectively). After adjusting for age, gender, extent of resection, adjuvant treatment and histological diagnosis, a Low triiodothyronine syndrome was not associated with 5-year mortality (p-values ≥.296). Lower total and free triiodothyronine concentration was associated with greater 5-year all-cause (HR=.038; 95%CI [.976-.999], p=.038 and HR=.538; 95%CI [.328-.882], p=.014, respectively) and brain tumor related (HR=.983, 95%CI [.968-.997], p=.02 and HR=.410; 95%CI [.216-.780], p=.007, respectively) mortality in all patients adjusting for age, gender, extent of resection, adjuvant treatment and histological diagnosis.

The Low triiodothyronine concentration below the reference range syndrome was associated with 2-fold increased risk of 1-year (p=.038), 2-year (p=.018) and 5-year (p=.008) mortality in high-grade and low-grade patients and in high grade glioma patients only, independent of age, gender, extent of resection, adjuvant treatment and histological diagnosis (Table 4).

**Table 4:** Association of low total triiodothyronine concentrations (<70 ng/dl) with mortality of glioma patients (Hazard ratio, 95% Confidence interval, p-value)

	1 year mortality	2 year mortality	5 year mortality
All glioma patients ^A^	2.394 (1.051-5.451), .038	2.203 (1.145-4.238), .018	2.338 (1.244 – 4.393), .008
High-grade glioma patients^B^	2.394 (1.051-5.451), .038	2.086 (1.078-4.033), .029	2.197 (1.160-4.163), .016

^A^ Multivariate analyses adjusted for age, gender, extent of resection, adjuvant treatment and histological diagnosis (enter method).

^B^ Multivariate analyses adjusted for age, gender, extent of resection and adjuvant treatment (enter method).

## DISCUSSION

The Low triiodothyronine syndrome was frequently observed in brain tumor patients, with the greatest prevalence in high-grade glioma patients. Lower triiodothyronine concentrations were associated with worse functional and cognitive status. The Low triiodothyronine syndrome was associated with a 3-fold increased risk of unfavorable discharge outcomes independent of age, gender and histological brain tumor diagnosis. The Low triiodothyronine syndrome was associated with shorter survival of glioma patients.

Nearly two thirds of primary brain tumor patients had reduced triiodothyronine concentrations. The prevalence of the Low triiodothyronine syndrome was the highest in high-grade glioma patients. In a previous study of 90 patients with primary and metastatic brain tumors, we found that 38% of patients had reduced triiodothyronine concentrations before surgery, and that the prevalence of the Low triiodothyronine syndrome increased to 54% after surgery [15]. Greater prevalence rate of the Low triiodothyronine syndrome in the present study can be explained by the greater number of patients with malignant gliomas (21% vs. 13%) included in the present cohort. Two smaller studies reported lower triiodothyronine concentrations in patients with brain tumors relative to healthy controls [13, 14]. Our findings suggest that greater reduction of triiodothyronine concentration might reflect more aggressive brain tumor biology and greater health status impairment imposed by brain tumor. High prevalence of the Low triiodothyronine syndrome was also reported in other critical illnesses, such as sepsis (65%) [17] and severe traumatic brain injury (60%) [8]. Impaired peripheral tissue 5’-deiodination and reduced hypothalamic and pituitary TRH and TSH secretion are the chief mechanisms responsible for development of the Low triiodothyronine syndrome [6, 9]. We found that triiodothyronine/thyroxine ratio was lower and rT3/thyroxine ratio was greater in patients with high-grade gliomas relative to patients with other diagnoses, suggesting suppressed of 5’ deiodinase activity in malignant glioma patients. Further studies should attempt to elucidate biological mechanisms responsible for impaired thyroid hormone metabolism in brain tumor patients.

The association of lower triiodothyronine concentrations with suboptimal functional and cognitive health status is new to the literature. In previous studies, lower triiodothyronine concentrations were associated with worse health-related quality of life [18], and with greater depressive and anxiety symptom severity [15] of brain tumor patients independently from histological diagnosis, patients’ age, gender and functional status. These findings suggest that thyroid impairment should be suspected in brain tumor patients presenting with cognitive, mental and functional complaints. Thyroid hormone replacement therapy was shown to improve cognitive functioning in hypothyroid patients [19] and in aneurysmal subarachnoid hemorrhage survivors [20]. However, whether thyroid hormone replacement therapy can improve health status in brain tumor patients remains to be seen.

Low total triiodothyronine concentration before surgery was associated with a nearly 3-fold increased risk for unfavorable outcomes at discharge, and this association was independent of patients’ age, gender and histological brain tumor diagnosis. These findings agree with previous data showing 8-fold greater risk for poor discharge outcomes in brain tumor patients with reduced free triiodothyronine concentrations [15]. An association of lower triiodothyronine concentrations with worse hospital discharge outcomes was previously reported in stroke [21] aneurysmal SAH [22] patients. Our findings imply that brain tumor patients with low preoperative triiodothyronine concentrations should be considered at increased risk for poor discharge outcomes.

For the first time in the literature we have documented that the Low T3 syndrome is associated with shorter survival in brain tumor patients. Reduced triiodothyronine concentrations were also associated with greater mortality, suggesting dose-response association between triiodothyronine concentrations even with the normal range and prognosis of brain tumor patients. In malignant glioma patients who had the lowest preoperative tri-iodothyronine concentrations relative to other brain tumor patients, preoperative Low triiodothyronine syndrome was associated with a 2-fold greater 5-year mortality risk, and this association was independent of patients’ age, gender, extent of resection and adjuvant therapy. The latter findings suggest the Low T3 syndrome can help to identify malignant glioma patients who are at the greatest risk for poor prognosis. Previous studies also documented that the Low triiodothyronine predicts greater mortality risk of stroke [23] and lung cancer patients [12] independently of clinical disease severity. Reduced triiodothyronine concentration should be considered an independent prognostic biomarker of shorter survival in brain tumor patients.

There remains an ongoing debate in the literature as to whether the Low triiodothyronine syndrome should be treated in critically ill patients [6, 24]. Two observational studies have reported improved survival of malignant glioma patients treated with large doses of triiodothyronine possibly due to increased brain tumor radiosensivity associated with triiodothyronine administration [25, 26]. It was also documented that triiodothyronine promotes re-differentiation of glioma cells and suppresses proliferation of high-grade glioma cells [10]. We believe that there is an urgent need for clinical trials aiming to investigate whether the treatment of the Low triiodothyronine syndrome can improve the prognosis of patients suffering from these devastating disorders.

Our study has limitations. Heterogeneous sample in terms of histological diagnosis prevented from investigation of the prognostic value of the Low triiodothyronine syndrome in more homogenous subgroups of brain tumor patients. Due to absence of long-term cognitive function assessment we were not able to evaluate the association of thyroid hormones with patient-oriented outcomes. On the other hand, large sample size, long follow-up period and in-depth assessment of HPT-axis functioning are the major advantages of the study.

In conclusion, the Low triiodothyronine syndrome is a common complication in brain tumor patients that is associated with poor functional and cognitive health status, and with increased risk for unfavorable discharge outcomes. Lower triiodothyronine concentrations are associated with greater 5-year mortality risk independent of age, gender, extent of resection, adjuvant treatment and histological diagnosis. In glioma patients, the Low T3 syndrome is associated with shorter survival independently of age, gender, extent of resection, adjuvant treatment and histological diagnosis. There is a need to evaluate whether treatment of the Low triiodothyronine syndrome can improve the prognosis of brain tumor patients.

## MATERIALS AND METHODS

Institutional review board approval for the study was obtained. All participants provided informed consent. Consecutive adult patients admitted for brain tumor surgery at the Department of Neurosurgery of Lithuanian University of Health Science in a period from January, 2010 until September, 2011, were considered for this prospective observational cohort study. Patients included in the pilot study^12^ were not included in this report. Two-hundred and seventy eight patients were identified and invited to participate in the study. However, 48 (12%) patients were excluded because they had missing blood samples (n=30) or were diagnosed with metastatic brain tumors (n=11) and other intracranial lesions (n=7), leaving a final sample of 230 patients (70% women; median age 58[21] years).

On admission, patients were evaluated for demographic characteristics and previous brain tumor treatments. In addition, 105 (46%) patients were evaluated for functional status (Barthel index; BI [27]) and 94 (41%) patients were evaluated for cognitive status (Mini Mental State Examination; MMSE) [28]. Patients were considered as having unfavorable functional status and unfavorable cognitive status if their BI and MMSE scores were <90 and <24, respectively.

Blood samples for evaluation of the HPT-axis hormone concentrations were obtained within two days of admission and before brain tumor surgery. Clinical outcomes at discharge were evaluated using the Glasgow outcome scale (GOS) [29]. Patients were considered to have unfavorable outcomes if their GOS score ranged between 1 (death) and 3 (severe disability). GOS scores of 4 (moderate disability) and 5 (low disability) were considered as favorable outcomes.

Mortality data were collected from the national death registry. Deaths that occurred between the study entry date and November, 2015 were considered for the analyses. Causes of death coded as C70-71, D32, D35.2, D43 or D44.3 were classified as brain tumor associated deaths [30].

### HPT-hormone assessment

Blood samples were drawn before breakfast, were rapidly centrifuged and serum was frozen at -40°C. Serum concentrations of free triiodothyronine, total triiodothyronine, free thyroxine, total thyroxine, thyroid stimulating hormone (TSH) (Tosoh AIA 600/21/1800) and reversed triiodothyronine (rT3) (ZENTECH; Belgium) were assessed by radioimmunoassay method. The reference values were as follows: free triiodothyronine, 2-4 pg/ml; total triiodothyronine, 70-170 ng/dl; free thyroxine, 7-17 pg/ml; total thyroxine, 4.5-12 μg/dl; TSH, 0.25 – 4.5 μIU/ml; and rT3, 0.09-0.35 ng/ml.

On the basis of free and total triiodothyronine laboratory reference ranges, patients were determined as having low free triiodothyronine concentrations (<2 pg/ml) and low total triiodothyronine concentrations (<70 pg/ml), respectively.

### Statistical analysis

Data were analyzed with SPSS 17.0 for Windows. All thyroid hormone serum concentrations were not normally distributed therefore non-parametric tests were used.

First, we investigated thyroid hormone serum concentrations as a function of histological brain tumor diagnosis by performing Kruskal-Wallis and Chi-squared tests. The association of MMSE and BI scores with HPT-axis hormone concentrations was evaluated by the Spearman correlation and binary regression analyses. Significant associations in univariate regression analyses were subsequently adjusted for age, gender and histological diagnosis (enter method).

Next, we investigated the association of discharge GOS score with HPT-axis hormone concentrations by performing Spearman correlation analyses and binary logistic regression analyses with poor discharge outcome (GOS score ≤3) as dependent variable and low triiodothyronine (free and total) concentrations as independent adjusting for age, gender and histological diagnosis (enter method).

Finally, we investigated the association of the Low triiodothyronine syndrome with 5-year overall and brain tumor survival in an all patients and in glioma patients by using Kaplan-Meier and Cox regression analyses adjusting for age, gender, extent of resection, adjuvant therapy and histological diagnosis. In regression analyses, age (in years) was entered as a continuous variable, and gender (men=1 or women=0), brain tumor histological diagnosis (high-grade glioma=1; low-grade glioma=2; meningioma=3; pituitary adenoma=4; acoustic neuroma=5; or other histological diagnosis=6), extent of resection (gross total resection=1 or subtotal resection /biopsy=0), and adjuvant chemotherapy or radiotherapy (yes=1 or no=0) were treated as categorical variables.
